# The Prognostic Value of Alpha-Fetoprotein Response for Advanced-Stage Hepatocellular Carcinoma Treated with Sorafenib Combined with Transarterial Chemoembolization

**DOI:** 10.1038/srep19851

**Published:** 2016-02-02

**Authors:** Lei Liu, Yan Zhao, Jia Jia, Hui Chen, Wei Bai, Man Yang, Zhanxin Yin, Chuangye He, Lei Zhang, Wengang Guo, Jing Niu, Jie Yuan, Hongwei Cai, Jielai Xia, Daiming Fan, Guohong Han

**Affiliations:** 1 Department of Liver Disease and Digestive Interventional Radiology, Xijing Hospital of Digestive Diseases, Fourth Military Medical University, Xi’an, China; 2 Department of Medical Statistics, Fourth Military Medical University, Xi’an, China; 3 Xijing Hospital of Digestive Diseases & State Key Laboratory of Cancer Biology, Fourth Military Medical University, Xi’an, China

## Abstract

This retrospective cohort study aimed to evaluate the prognostic value of the alpha-fetoprotein (AFP) response in advanced-stage hepatocellular carcinoma (HCC) patients treated with sorafenib combined with transarterial chemoembolization. From May 2008 to July 2012, 118 HCC patients with baseline AFP levels >20 ng/ml treated with combination therapy were enrolled. A receiver operating characteristic curve was used to generate a cutoff point for AFP changes for predicting survival. The AFP response was defined as an AFP decrease rate [ΔAFP(%)] greater than the cutoff point. The ΔAFP(%) was defined as the percentage of changes between the baseline and the nadir values within 2 months after therapy. The median follow-up time was 8.8 months (range 1.2–66.9). A level of 46% was chosen as the threshold value for ΔAFP (sensitivity = 53.7%, specificity = 83.3%). The median overall survival was significantly longer in the AFP response group than in the AFP non-response group (12.8 vs. 6.4 months, *P* = 0.001). Multivariate analysis showed that ECOG ≥ 1 (HR = 1.95; 95% CI 1.24–3.1, *P* = 0.004) and AFP nonresponse (HR = 1.71; 95% CI 1.15–2.55, *P* = 0.009) were associated with increased risk of death. In conclusion, AFP response could predict the survival of patients with advanced-stage HCC at an early time point after combination therapy.

Hepatocellular carcinoma (HCC) is the sixth most common cancer worldwide with more than 800,000 newly diagnosed cases per year^1^. It is the second most common cause of cancer-related death in the world^2^. A large proportion of HCC patients are diagnosed at an intermediate or advanced stage beyond curative treatments. Based on the Barcelona Clinic Liver Cancer (BCLC) staging system, transarterial chemoembolization (TACE) and sorafenib are the standard treatments for intermediate and advanced-stage HCC, respectively^3^,^4^. Because Sorafenib may improve the efficacy of TACE therapy by decreasing post-TACE angiogenesis, sorafenib combined with TACE has been considered to be a promising therapy^5^,^6^,^7^. Although the preliminary results of the first randomized controlled (SPACE) trial were disappointing as the time to progression (TTP) failed to show a significant difference, we consider that the reason for the failure may lie in the study design. The other trials in this field are still underway. The answer regarding the superiority of combination therapy will be provided by the results of these trials in the future.

Currently, radiological imaging evaluation is widely used for the prognostic assessment of HCC. The Response Evaluation Criteria in Solid Tumors (RECIST) focuses on whole-tumor shrinkage^8^. The modified RECIST (mRECIST) criteria measure the change of the tumor necrotic area. However, radiological imaging evaluation has several limitations^9^. First, it is challenging to measure tumor size when the tumor grows in a diffuse pattern. Second, radiological imaging evaluation is a relatively subjective assessment and lacks inter-observer reproducibility^10^. Third, our previous studies showed that RECIST and mRECIST criteria fail to predict survival at an early time point^11^. Therefore, alternative methods to estimate treatment efficacy are needed.

Alpha-fetoprotein (AFP) is a glycoprotein that is secreted in approximately 70% of HCC^12^. As the most common biomarker of HCC, AFP has confirmed its value in screening and diagnoses in multiple studies^13^. Recently, several studies unanimously suggested that the AFP response was associated with longer overall survival (OS) in HCC patients after locoregional treatment modalities or systematic chemotherapy^14^,^15^. However, the prognostic value of the AFP response in patients with advanced-stage HCC who are treated with sorafenib combined with TACE remains unclear.

The aim of this study was to evaluate the prognostic value of the AFP response in patients with advanced HCC who were undergoing treatment with sorafenib combined with TACE and to explore the correlation between the AFP response and a radiological evaluation from an early time point.

## Materials and Methods

All HCC patients consecutively admitted to our department between May 2008 and June 2012 who were treated with a combination therapy of sorafenib and TACE were retrospectively considered in our study. The inclusion criteria were as follows: 1) an age ≥18 years old, 2) an interval between sorafenib and TACE of ≤60 days, 3) an Eastern Cooperative Oncology Group (ECOG) performance status score ≤2, 4) a Child-Pugh A or B (≤7), and 5) no other molecular target agents. The exclusion criteria were as follows: 1) main portal vein invasion,2) concurrent malignancy,3) an absence of a repeat AFP measurement within 2 months after treatment initiation,4) a baseline AFP < 20 ng/ml, and 5) poor compliance. The diagnosis of HCC was based on the American Association for the Study of Liver Disease (AASLD) criteria^16^. Histology was needed only in case of diagnostic uncertainty. OS was measured from the beginning of combination therapy to the date of death or the last follow-up. The requirement to obtain informed consent was waived. The study protocol was approved by the ethics committees of Xijing Hospital. All the methods used in this study were carried out according to the approved guidelines.

### Treatment and follow-up

The patients received sorafenib at an initial dose of 400 mg twice daily. Later, the dose of sorafenib was modified based on the degree of adverse events (AEs). AEs were assessed according to the National Cancer Institute Common Terminology Criteria for Adverse Events version 4.0. In our clinical practice, patients continue sorafenib treatment if the AEs can be safely controlled. TACE was performed using 10–50 mg doxorubicin mixed with 5–20 mg lipiodol. Gelatin foam was injected until the tumor-feeding vessels were completely obstructed. TACE procedures were repeated according to the radiological response^5^. Combined therapy was defined as an interval between sorafenib and TACE of less than 60 days, regardless of the order of the two treatments. Standard follow-up evaluations, including contrast-enhanced computer tomography (CT) scans and laboratory assessments, were performed during weeks 4 and 8 after the initiation of treatment and every 8 weeks thereafter. The end of the follow-up period was either death or December 31^st^ 2014.

### AFP evaluation

The serum AFP concentration was measured at baseline (before the initiation of combined therapy) and at every follow-up visit using an electro chemiluminescence immunoassay (ElecsysCobas e601, Roche). The AFP variation rate (ΔAFP) was defined as the percentage of change between the baseline and the nadir within 1–2 months after combination therapy.


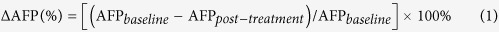


The AFP response was defined as an ΔAFP(%) greater than the cutoff point (ΔAFP(%) > cutoff point), whereas AFP non-response was defined as an AFP decrease rate less than the AFP variation cutoff point (ΔAFP(%) < cutoff point). The researcher who extracted the AFP data was blinded to the survival outcome.

### Radiological evaluation and definitions

Radiological imaging assessments were performed with contrast-enhanced spiral computed tomography (CT) at baseline (before the initiation of combined therapy) and at every follow-up visit after combined therapy. The RECIST and mRECIST criteria were used for radiological evaluation. The treatment responses were blindly assessed by three experienced clinicians (Yan Zhao, JiaJia and Wei Bai). In cases of discrepancies, the images were jointly reviewed by all of the clinicians, and a consensus decision was reached. If the patients were evaluated as having a complete response (CR) or a partial response (PR) within 2 months after combination therapy, these individuals were considered to be responders. If the patients were evaluated as having stable disease (SD) or progressive disease (PD), these individuals were considered to be non-responders^17^.

### Statistical analyses

Continuous variables were presented as median values with ranges, and categorical variables were presented as frequencies with percentages. A receiver operating characteristic (ROC) curve was used to generate a cutoff point for AFP changes that predicted survival. For the area under the curve, a cutoff point with the highest sum of sensitivity and specificity was chosen as the most discriminative value of the AFP response for predicting survival. This statistic may range from 0 to 1, and cutoff points with a c-statistic >0.7 are generally considered useful^18^. A Mann-Whitney U test was used to compare continuous variables, whereas a Chi-squared test was used to compare categorical variables between the AFP response and non-response groups. The Κ coefficient was used to measure the inter-method concordance of the radiological response and the AFP response. OS time was assessed by Kaplan-Meier methods, and the survival difference between groups was estimated by the log-rank test. Patients lost to follow-up or alive at the end-of-observation date were censored. Univariate and multivariate Cox regression analyses were used to test the prognostic factors of OS. Variables with a *P* value < 0.1 in the univariate analysis were included in the multivariate analysis. Statistical analyses were performed using SPSS version 16.0 (SPSS, Inc., Chicago, IL, USA). A two-sided *P* value < 0.05 was considered to be statistically significant.

## Results

### Patient characteristics and treatment

**Figure 1:**
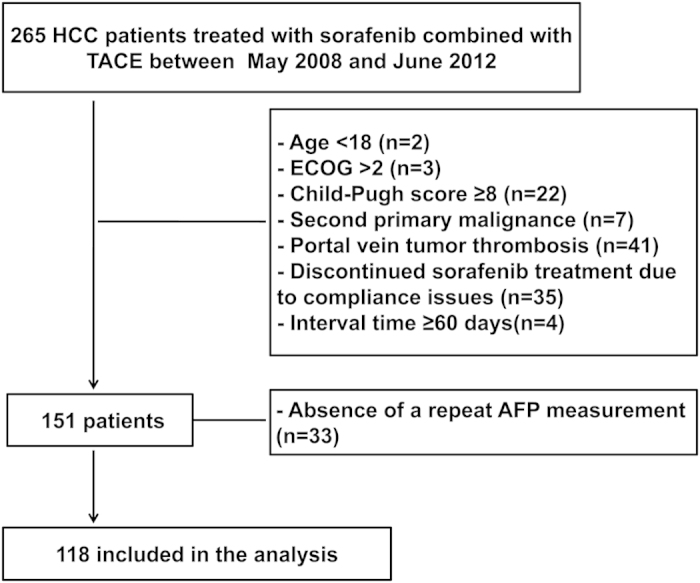
Enrollment and outcomes.

A total of 118 patients with unresectable HCC were included in our study (Fig. 1). The median age was 48 years (range, 23–75 years). Most patients were male (86.4%) and had hepatitis B virus infection (89%), Child-Pugh class A (90.7%), an ECOG performance status of 1–2 (74.6%), and BCLC stage C (83.9%). In total, 47 (66.9%) patients had branch portal vein tumor thrombosis (PVTT). Extrahepatic spread was observed in 45 (38.1%) patients, mainly in the abdominal lymph nodes (46.5%), the lungs (39.5%) and skeleton (16.3%) (Table 1). The median number of sessions of TACE was 2 (range, 1–12), the median time taking sorafenib was 6.6 months (range 0.3–66.9 months) and the median interval between sorafenib and TACE was 3 days (range 0–55 days). The interval was <7 days for 108 patients (91.5%), was <15 days for 8 patients and 15–55 days for 2 cases. The median baseline AFP level was 1821.5 ng/ml (range 20.7–121000 ng/ml), 25 (21.2%) patients had <200 ng/ml and 93 (78.8%) patients had ≥200 ng/ml.

### Survival analysis

**Figure 2:**
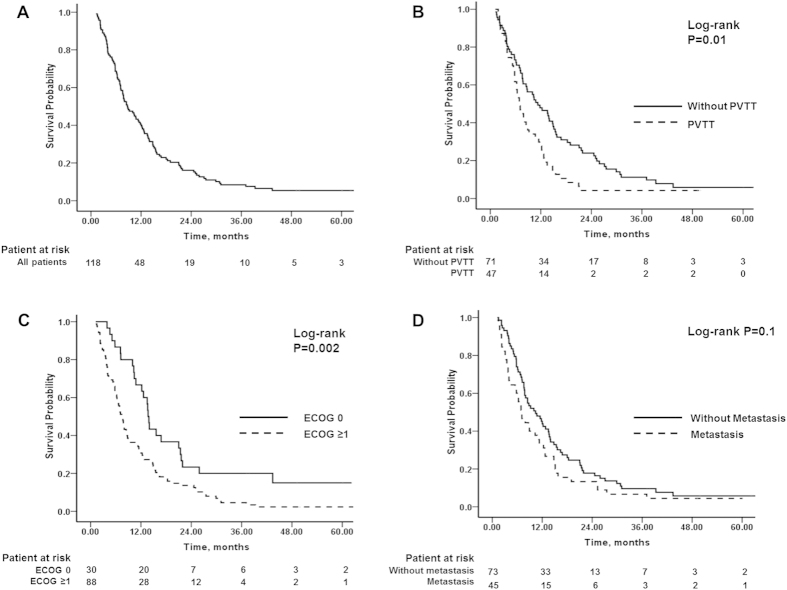
The Kaplan-Meier analysis of overall survival. (**A**) Overall survival. (**B**) A comparison of survival according to portal vein thrombosis. (**C**) A comparison of survival according to the ECOG score. (**D**) A comparison of survival times according to extrahepatic metastasis.

The median follow-up time was 8.8 months (range, 1.2–66.9). By the end of follow-up, 111 out of 118 patients (94.1%) died and 7 (5.9%) survived. The overall median survival was 8.7 months (95% CI, 6.5–10.9) (Fig. 2A). The median OS was 11.3 months (95% CI, 6.9–15.8) in the patients with PVTT and 8.7 months (95% CI, 5.1–8.9) in the patients without PVTT (*P* = 0.011) (Fig. 2B). The median OS of the patients with ECOG 0 was longer than that of patients with ECOG ≥ 1 (13.7 months vs. 7.6 months, *P* = 0.002) (Fig. 2C). The difference in OS between patients with extrahepatic metastasis and those without metastasis was not significant (10.4 months vs. 7 months, *P* = 0.1) (Fig. 2D).

### A comparison between AFP response and non-response groups

**Figure 3:**
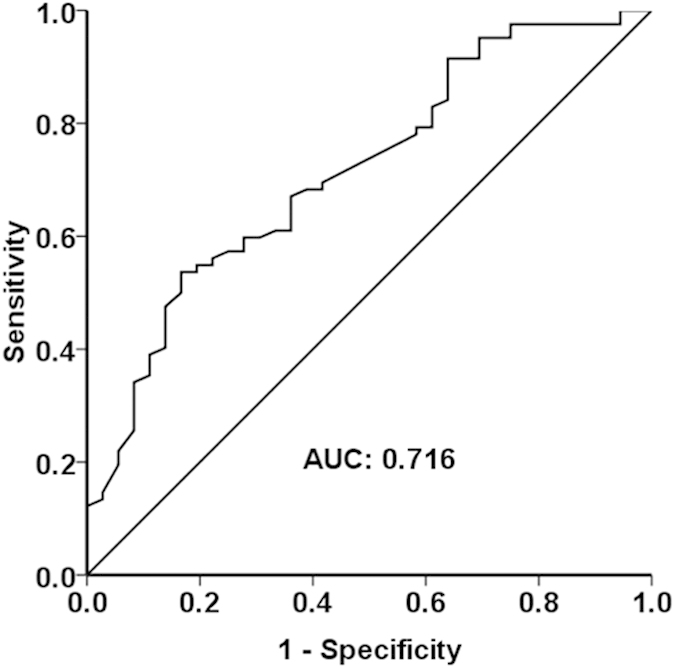
The ROC curve for AFP values and survival.

The median time from the baseline treatment to AFP follow-up was 1.4 months (range 0.4–2.0). The area under the ROC curve (c-statistic) for predicting survival was 0.716 (Fig. 3). The most discriminative value of the ΔAFP(%) for predicting survival was 46%. This cutoff point had a sensitivity of 53.7% and a specificity of 83.3%.

**Figure 4:**
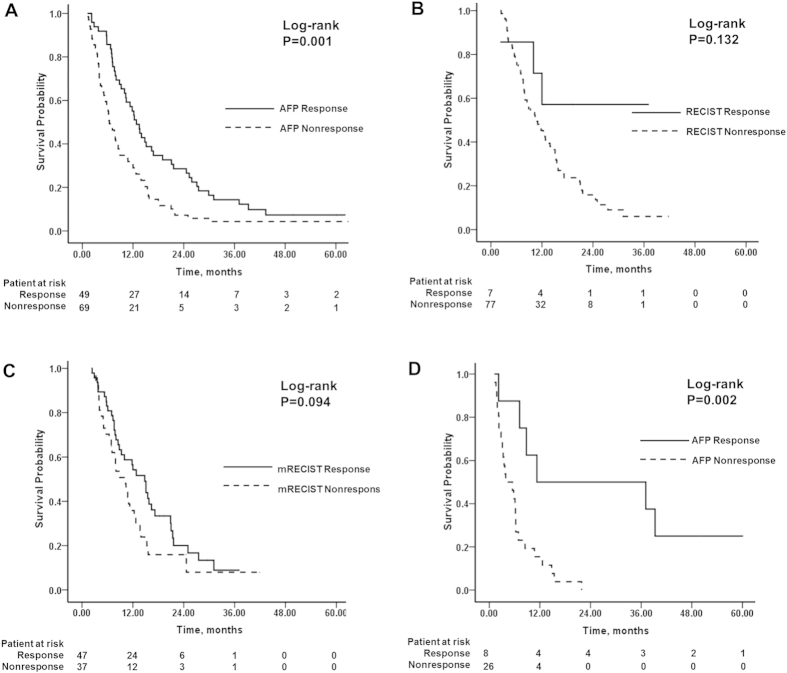
The Kaplan-Meier analysis of overall survival. (**A**) A comparison between the AFP response and nonresponse groups in the entire cohort; (**B**) A comparison between RECIST response and nonresponse groups in 84 patients with radiological response; (**C**) A comparison between mRECIST response and nonresponse groups in 84 patients with radiological response; (**D**) A comparison between the AFP response and nonresponse groups in 34 patients without radiological evaluation.

In this study, 49 (41.5%) patients with ΔAFP(%) > 46% were classified into the AFP response group and 69 (58.5%) patients with ΔAFP(%) < 46% were classified into the non-response group. Most baseline clinical characteristics were similar between the AFP response and non-response groups, but the proportion of males was higher in the AFP response group than in the non-response group (Table 1). The median OS was significantly longer in the AFP response group (12.8 months, 95% CI 10.2–15.3) than in AFP non-response group (6.4 months, 95% CI4.7–8.1) (*P* = 0.001) (Fig. 4A). Multivariate analysis showed that ECOG ≥ 1 (HR = 1.95; 95% CI 1.24–3.1, *P* = 0.004) and AFP nonresponse (HR = 1.71; 95% CI 1.15–2.55, *P* = 0.009) were associated with increased risk of death (Table 2).

### The correlation between AFP response and radiological evaluation

Of the 118 patients, 84 (71.2%) were properly evaluated according to both RECIST and mRECIST criteria. Survival was of insufficient time to carry out contrast-enhanced CT scans in 1 patient, 3 patients did not have a complete imaging examination due to clinical deterioration, 10 patients had non-measurable diffused tumor lesions in the liver, and 20 patients did not have completely preserved follow-up image data. The median time for assessing radiological imaging response was 1.2 months (range, 0.7–2.0 months). The rates of CR, PR, SD and PD were 0, 7 (8.3%), 66 (78.6%) and 11 (13.1%), respectively, according to the RECIST criteria, and 24 (28.6%), 23 (27.4%), 30 (35.7%) and 7 (8.3%), respectively, according to the mRECIST criteria. The response rates (CR and PR) and nonresponse rate (SD and PD) were 8.3% and 91.7% according to the RECIST criteria and 56% and 44% according to the mRECIST criteria, respectively. With RECIST criteria, the median survival value of response group was not obtained because too few patients (n = 7) were classified into this group and 4 patients were censored. However, there was no difference between the response and nonresponse groups (*P* = 0.132) (Fig. 4B). With mRECIST criteria, the survival difference was not statistically significant between the response and nonresponse groups [14.8 months (95% CI 10.9–18.7) vs. 10.3 months (95% CI 6.8–13.8), *P* = 0.075] (Fig. 4C). Multivariate analysis showed that both the RECIST (HR = 2.2; 95% CI 0.9–5.6, *P* = 0.094) and mRECIST (HR = 2; 95% CI 0.9–2.2, *P* = 0.160) criteria were not independent predictors of overall survival. The outcomes of both the radiological assessment and AFP response are shown in Table 3. The patient evaluation in every response category was markedly different between the RECIST criteria and the AFP response (Κ = 0.077), whereas the majority of patients were classified into the same response categories when assessed using the mRECIST criteria and the AFP response. However, the agreement was still weak between the mRECIST criteria and the AFP response (Κ = 0.383).

Of the 34 patients without radiological evaluation, 8 and 26 patients were in the AFP response and AFP non-response groups, respectively. The median OS was significantly longer in the AFP response group than in the AFP non-response group (11.3 months vs. 3.9 months, *P* = 0.002) (Fig. 4D).

## Discussion

Because AFP assessment is a simple and reproducible method to for the evaluation of the efficacy of combination treatment, our study demonstrates the feasibility of using the dynamic trend of AFP as an early biomarker for predicting survival outcomes after combination therapy in advanced HCC patients.

AFP is a well-established tumor marker for screening and diagnosing HCC, and the AFP level appears to be associated with the prognosis of HCC patients^19^. Previous studies demonstrated that an elevated AFP level would decrease in HCC patients after hepatic resection and would rebound in cases of HCC recurrence^20^. Recently, the AFP response has been reported to be a significant prognostic factor in HCC patients treated with different locoregional modalities or systemic chemotherapy^14^,^15^,^21^. To our knowledge, the current analysis is the first exploration of the potential prognostic value of the AFP response in HCC patients treated with sorafenib combined with TACE. And our study population was mainly consisted of advanced stage HCC patients, which was different from previous report. The major findings of this study were as follows: 1) the adaptive AFP variation cutoff point to predict prognosis was a 46% reduction, 2) the AFP response (a decline of more than 46% from baseline within 2 months after the initiation of combination therapy) was associated with longer OS in patients with advanced-stage HCC who were treated with sorafenib in combination with TACE, and 3) the AFP response could predict the overall survival at an earlier time point compared to radiological assessment, particularly in circumstances in which radiological evaluation could not be performed.

In previous studies, the AFP response was defined as an AFP level that decreased by more than 20%, 30% or 50%^14^,^22^,^23^. However, the definition of the AFP response mostly originated from personal clinical experiences or speculation but not from statistical analyses. In contrast, we used a ROC curve to generate an adaptive AFP variation cutoff point (an AFP reduction of 46%) for the AFP response. More importantly, by using this cutoff point the AFP response group had significantly longer survival than the AFP nonresponse group, and it was an independent predictor for overall survival. Thus, the AFP level could be incorporated into the algorithm for assessing the prognosis of HCC patients. Additionally, it should be noted that patient selection in previous studies was different from ours. In previous studies, patients with baseline AFP < 100 ng/ml or <200 ng/ml were excluded to differentiate from other benign liver diseases^14^,^21^. Thus, the conclusions of these studies were suitable only for patients with a relatively high baseline AFP level. In contrast, our inclusion criteria were relatively wider, only patients with a baseline AFP < 20 ng/ml were excluded from our study because not all HCC patients have an elevated AFP level.

Radiological evaluations, such as those based on RECIST and mRECIST criteria responses, have been widely used in the prognostic assessment of HCC^17^,^24^. Radiological response has also been established to correlate with the pathological response^25^,^26^. However, in the current study, both the RECIST and mRECIST assessment within 2 months after treatment were not independent predictors of overall survival. Additionally, the agreement between radiological assessment and the AFP response was weak regardless of whether the RECIST or mRECIST criteria were used, though a majority of patients were classified into the same response categories when assessed using the mRECIST criteria and the AFP response. These results were consisted with our previous study that showed that the earliest time to evaluate the response to combination therapy was 3 months^11^. This result could be explained by the reality that the baseline tumor burden in Chinese patients is higher than those reported in western countries. Only one TACE session may not be efficient enough to achieve complete tumor response. Moreover, the study by Georgiades *et al*. showed that initial nonresponders after the first TACE session could obtain prolonged survival from further treatment^27^. Therefore, under these circumstances, radiological assessment could not be used as an early predictor of overall survival. Additionally, our study demonstrated that the AFP response could predict the prognosis of these patients in the absence of a radiological evaluation, especially in patients with diffuse malignant tumors that could not be evaluated by radiological criteria. Hypovascular or diffusely infiltrative tumor patterns are often present in real-world clinical settings^22^. Establishing a correlation between AFP and treatment efficacy has the potential to help assess treatment response in clinical practice when the standard imaging findings are equivocal. Another potential advantage of the AFP assessment would be reducing the cost burden of repeat radiological scans.

Several limitations of this study should be recognized. First, this was a retrospective study with a relatively small number of patients. A potential bias may exist because not all the patients had follow-up AFP assessments within 2 months after treatment and consequently only the patients with complete follow-up information were included in the analysis. Further well-design prospective studies with large sample sizes are needed to confirm the prognostic value of the AFP response. Second, the serum AFP concentration might be influenced by hepatitis, cirrhosis and liver cell necrosis. Not all HCC patients have a significantly elevated AFP level at baseline, and patients with viral hepatitis and other benign liver diseases incidentally do have an elevated AFP level^28^,^29^. An AFP reduction might also be induced not only by treatment for HCC but also by antiviral or anti-fibrosis therapy. Unfortunately, the related data were lacking because we did not collect follow-up information about these types of therapies. Third, the 46% cut-off point was based on this study cohort that mainly consisted of advanced stage HCC patients. Its application in patients with intermediate stage HCC requires further validation.

In conclusion, our study suggested that the AFP response could predict overall survival in advanced-stage HCC patients at an early time point after the treatment of sorafenib combined with TACE. Further prospective studies are necessary to validate the prognostic effect of a decline of 46% as an accurate AFP variation cutoff point.

## Additional Information

**How to cite this article**: Liu, L. *et al*. The Prognostic Value of Alpha-Fetoprotein Response for Advanced-Stage Hepatocellular Carcinoma Treated with Sorafenib Combined with Transarterial Chemoembolization. *Sci. Rep*. **6**, 19851; doi: 10.1038/srep19851 (2016).

## Figures and Tables

**Table 1:** Baseline demographics and clinical characteristics.

Variables	All patients (n = 118)	AFP response (n = 49)	AFP non-response (n = 69)	*P value*	
Age (y)					
Median (Range)	48 (23–75)	49 (30–74)	47 (23–75)	0.564	
Sex					
Male/Femal - No. (%)	102 (86.4%)/16 (13.6)	38 (77.6%)/11 (22.4%)	64 (92.8%)/5 (7.2%)	0.017	
Etiology					
HBV/HCV/Other - No. (%)	105 (89%)/2 (1.7%)/11 (9.3%)	41 (83.7%)/2 (4.1%)/6 (12.2%)	64 (92.8%)/0 (0%)/5 (7.2%)	0.146	
Child-Pugh class					
A/B - No. (%)	107 (90.7%)/11 (9.3%)	44 (89.8%)/5 (10.2%)	63 (91.3%)/6 (8.7%)	1	
ECOG					
0/1–2 - No. (%)	30 (25.4%)/88 (74.6%)	16 (32.7%)/33 (67.3%)	14 (20.3%)/55(79.7%)	0.139	
BCLC stage					
B/C - No. (%)	19 (16.1%)/99 (83.9%)	11 (24.5%)/38 (75.5%)	8 (13%)/61 (87%)	0.135	
Disease burden					
PVTT					
No/yes - No. (%)	71 (33.1%)/47 (66.9%)	33 (67.3%)/16 (32.7%)	38 (55.1%)/31 (44.9%)	0.189	
Extrahepatic spread					
No/Yes - No. (%)	73 (61.9%)/45 (38.1%)	33 (67.3%)/16 (32.7%)	40 (58%)/29 (42%)	0.256	
Baseline tumor size (cm)					
Median (Range)	10.8 (2.9–25.3)	10.5 (3.5–22.1)	11.7 (2.9–25.3)	0.325	
No. of HCC nodules					
1/≥2/Diffused - No. (%)	86 (72.9%)/22 (18.6%)/10 (8.5%)	39 (79.6%)/8 (16.3%)/2 (4.1%)	47 (68.1%)/14 (20.3%)/8 (11.6%)	0.264	
Baseline AFP (ng/ml)					
<200/≥200 - No. (%)	25 (21.2%)/93 (78.8%)	12 (24.5%)/37 (75.5%)	13 (18.8%)/56 (81.2%)	0.459	
Liver biopsy					
Yes/No - No. (%)	19 (16.1%)/99 (83.9%)	9 (18.4%)/40 (81.6%)	10 (14.5%)/59 (85.5%)	0.573	
Ascites					
Yes/No - No. (%)	22 (18.6%)/96 (81.4%)	10 (20.4%)/39 (79.6%)	12 (17.4%)/57 (82.6%)	0.678	
Laboratory values, mean (range)					
Alanine aminotransferase (U/I)	57.7 (10–395)	62.9 (10–395)	54.1 (13–236)	0.355	
Aspartate aminotransferase (U/I)	77.6 (16–489)	74.1 (16–362)	80 (16–489)	0.639	
Total bilirubin, mg/dl	17.9 (6.9–54)	17.5 (7.1–362)	18.2 (6.9–38.4)	0.698	
Platelets/mm^3^	163 (31–511)	150.4 (47–362)	173 (31–511)	0.166	
International normalized ratio	1.1 (0.73–1.58)	1.1 (0.91–1.4)	1.1 (0.73–1.58)	0.682	
Albumin, g/dl	39.5 (29.4–75.6)	40.3 (32–75.6)	38.9 (29.4–52.9)	0.201	
Order of treatments					
Sorafenib before TACE/TACE before sorafenib/Meanwhile - No. (%)	52 (44.1%)/62 (52.5%)/4 (3.4%)	20 (40.8%)/28 (57.1%)/1 (2.1%)	32 (46.4%)/34 (49.3%)/3 (4.3%)	0.610	
Interval between TACE and sorafenib (d)					
Median (Range)	3 (0–55)	2 (0–40)	3 (0–55)	0.073	

HBV, hepatitis B virus; HCV, hepatitis C virus; ECOG, Eastern Cooperative Oncology Group; BCLC, Barcelona Clinc Liver Cancer; PVTT, portal vein tumor thrombosis; HCC, hepatocellular carcinoma; AFP, alpha-fetoprotein; TACE, transarterial chemoembolization.

**Table 2:** Univariate and multivariate analysis for overall survival*.

Variable	Univariate analysis			Multivariate analysis		
	HR	95% CI	*P* value	HR	95% CI	*P* value
Age (y)	1.003	0.986–1.020	0.720	–	–	–
Sex						
Female vs. male	0.920	0.541–1.564	0.758	–	–	–
Etiology						
Hepatitis infection vs. Other	0.749	0.398–1.410	0.370	–	–	–
Child-Pugh class						
B vs. A	0.943	0.490–1.814	0.861	–	–	–
ECOG						
2 vs. 0–1	2.208	1.294–3.180	0.002	1.952	1.239–3.076	0.004
PVTT						
Yes vs. No	1.658	1.124–2.447	0.011	1.398	0.934–2.093	0.103
Extrahepatic spread						
Yes vs. No	1.376	0.938–2.017	0.103	1.214	0.825–1.787	0.324
No. of HCC nodules						
≥2 + Diffused vs. 1	1.072	0.735–1.565	0.718			
Baseline AFP (ng/ml)						
≥200 vs 200	1	0.669–1.494	0.999	–	–	–
AFP change						
Nonresponse vs. response	1.863	1.268–2.738	0.002	1.710	1.147–2.551	0.009

HCC, hepatocellular carcinoma; HR, hazard ratio; CI, confidence interval; ECOG, Eastern Cooperative Oncology Group; PVTT, portal vein tumor thrombosis; AFP, alpha-fetoprotein; TACE, transarterial chemoembolization.

*To avoid effect of colinearity with variables, BCLC and ascites were not included in the model.

**Table 3:**
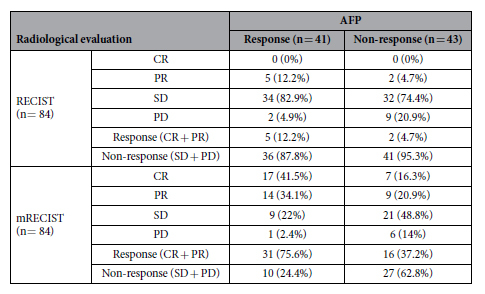
The correlation between the radiological evaluation and AFP assessment.

AFP, alpha-fetoprotein; RECIST, Response Evaluation Criteria in Solid Tumor; mRECIST, Modified Response Evaluation Criteria in Solid Tumor; CR, complete response; PR, partial response; SD, stable disease; PD, progressive disease.
